# Immunomodulatory effects of platelets on the severity of hand, foot, and mouth disease infected with enterovirus 71

**DOI:** 10.1038/s41390-020-0970-y

**Published:** 2020-06-09

**Authors:** Qianwen Li, Yimeng Wang, Wenyao Xue, Zhengying Bian, Yue Gao, Yu Zeng, Lei Tang, Tiejun Tang, Ye Tian, Wei Guo

**Affiliations:** 1 Jiangsu Key Laboratory of Druggability of Biopharmaceuticals, School of Life Science and Technology, China Pharmaceutical University, Nanjing, China; 2 Children’s Hospital of Nanjing Medical University, Nanjing, China

## Abstract

**Background:**

Enterovirus 71 (EV71) infection contributes to hand, foot, and mouth disease (HFMD) with severe neurogenic complications, leading to higher morbidity. In addition to their typical roles in coagulation, platelets could serve as essential immune regulatory cells to play a key role in the pathogenesis of this viral infection.

**Methods:**

Platelet parameters were measured using an automatic hematology analyzer. T-helper type 1 (Th1) and Th2 cells were analyzed by flow cytometry. The levels of cytokines and key transcription factors were determined.

**Results:**

The levels of platelet count and plateletcrit were positively associated with the severity of HFMD. Th1 and Th2 cells as well as their corresponding cytokines were increased in the severe group compared to the healthy volunteers. Moreover, the levels of platelets were negatively correlated with the level of interferon-γ (IFN-γ), but positively correlated with the frequency of Th1 cells. Coculture of platelets and naive CD4^+^ T cells showed that platelets from mild patients promote Th1 cell differentiation and IFN-γ secretion.

**Conclusions:**

Our study has shown for the first time that the distinct roles of platelets are responsible for the regulation of pathogenic CD4^+^ T cell differentiation and function in the pathogenesis of HFMD caused by EV71.

**Impact:**

- Our study has shown for the first time that the distinct roles of platelets are responsible for the regulation of pathogenic CD4^+^ T cell differentiation and function in the pathogenesis of HFMD caused by EV71.
- For the first time, we have discovered the role of platelets in children’s HFMD caused by EV71 infection, which may provide a better treatment for HFMD in the future.
- This article describes new discoveries in platelet immunity.

## Introduction

Enterovirus 71 (EV71),^1^ belonging to the family *Picornaviridae*, genus *Enterovirus* and species *Enterovirus A*, is one of the pivotal etiological factors for hand, foot, and mouth disease (HFMD).^2^ This disease is primarily prevalent in Asian countries, particularly in China.^3^ Clinical staging of EV71 infection is classified as eruption, nervous system involvement, early cardiopulmonary failure, cardiopulmonary failure, and recovery.^4^ As the brain stem is the main target of an EV71 attack, it can develop encephalomeningitis,^5,6^ encephalitis or myocarditis, and neurogenic pulmonary edema. To date, no effective drugs for EV71 infection are available because of the unclear pathogenesis of EV71 infection.^7^ It was reported that the expression of pattern recognition receptors (PRRs), including retinoic acid-inducible gene-1 and Toll-like receptors, and the inflammatory regulator mediator CC chemokine ligand 5 (CCL5) is related to the disease susceptibility and severity of EV71 infection.^8–11^ On the other hand, immune cell dysfunction and inflammation overactivation participate in the progression of HFMD.^12,13^ For example, our previous study found that intrinsic lymphocyte cell 1 abnormalities contribute to the development of severe HFMD by activating and cooperating cellular and humoral immunity. In addition, it was demonstrated that proinflammatory cytokine/chemokine profiles, including interleukin-1β (IL-1β), IL-6, IL-10, monocyte chemoattractant protein-I and interferon-γ-inducible protein-10, are significantly increased in children with severe HFMD.^14,15^ Moreover, the levels of platelet-activating factor, histamine, noradrenaline, and IL-4 increased significantly in an EV71 infection-induced pulmonary edema mouse model.^7^

Platelets derived from megakaryocytes are commonly classified as cellular mediators of hemostasis with essential roles in the progression of thrombosis and wound healing.^16^ Apart from this conventional view, it has been progressively evident from recent studies that platelets could influence all stages of immune responses,^17^ which are involved in the pathogenesis of various diseases, specifically viral infection. Because of the high expression of PRRs, platelets could promote the recognition of viruses and affect viral proliferation or clearance through the PRR-PAMP recognition module.^18^ It was suggested that platelets attached and internalized dengue virus through dengue virus-associated PRR-like heparan sulfate and DC-SIGN (dendritic cell-specific intercellular adhesion molecule-3-grabbing non-integrin).^19^

Platelets can regulate immune cells in two ways, which further affects the inflammatory response.^17^ Platelets store many proinflammatory and regulatory mediators in their α-particles and dense particles, which are rapidly released at the site of inflammation or tissue injury.^20^ Platelet-derived mediators include cytokines (IL-1α, IL-1β, and tumor growth factor-β1), chemokines (platelet factor 4 (PF4), CXCL4, and CCL3), immunomodulatory neurotransmitters (serotonin, dopamine, epinephrine, histamine, and γ-aminobutyric acid) and other low-molecular-weight mediators.^21^ In addition to the secretion of soluble factors, activated platelets also upregulate many integrins, adhesion molecules, and lectins, thus forming platelet–T cell aggregates.^22^ Fixed platelets are bridged by P-selectin to support the recruitment of lymphocytes at the inflammatory site. In addition, platelets can adhere to lymphocytes to form platelet–lymphocyte conjugates. Since platelets enhance the production of T cell cytokines by CD40-CD40L ligation, they can provide a direct way for platelets to communicate with CD4^+^ T cells. For example, in PF4^−/−^ mice, platelet-derived PF4 contributed to a limitation in Th17 cell differentiation, demonstrating a crucial role for platelets in T cell homeostasis.^23^ In addition, the activation and degranulation of platelets is responsible for the stimulation of Th1 and Th17 cells in the experimental autoimmune encephalomyelitis model of multiple sclerosis (MS).^24^ While it has been widely recognized that platelets serve as immune regulatory cells to affect the pathogenesis of virus infection,^23^ whether and how platelets impact the pathogenesis of EV71 infection remain to be explored. Moreover, according to clinical observations of hospitalized HFMD patients in our previous study, we found that the platelet count (PLT) is significantly increased in children with EV71 infection;^25^ however, the underlying mechanism of platelets in the initiation and exacerbation of inflammation is unclear.

In this study, we aimed to investigate whether and how platelets participate in the pathogenesis of EV71 infection. Our data show that the platelet level in children with EV71 infection is positively correlated with an increase in Th1 cells, but is negatively associated with the level of interferon-γ (IFN-γ) in severe viral encephalomeningitis (VEM) patients, suggesting that platelets may inhibit the function of Th1 cells in the development of VEM. In addition, through coculture in vitro, we found that platelets in mild patients could promote Th1 cell differentiation in a certain concentration range, but platelets in severe patients exhibited an inhibitory effect on Th1 cells. Based on our experimental data, platelets in severe patients could directly inhibit Th1 cell differentiation by several surface molecules CD40L, GPIbα, and P-selector (CD62P).

## Materials and methods

### Clinical samples and sample preparation

Our research was carried out in accordance with the World Medical Association Declaration of Helsinki. All subjects provided written informed consent documents, and this study was approved by the Ethical Committee of Children’s Hospital of Nanjing Medical University. Twenty-six healthy volunteers (*n* = 26, 18 M/8 F) and 53 EV71-infected HFMD children without drug treatment were recruited during outbreaks between January 2018 and December 2018 at the Affiliated Children’s Hospital of Nanjing Medical University. The cohort was divided into two groups based on the definitions of mild and severe HFMD. Mild group: patients with only mild symptoms, including fever, blisters on mouth, hand and feet, and with a good prognosis (*n* = 23, 18 M/5 F); severe group: patients with VEM, which was characterized by a high level of nucleated cell count (*n* > 30 × 10^6^/L) in the cerebrospinal fluid (CSF) (*n* = 30, 22 M/8 F). Clinical materials and samples were collected at admission. Sex- and age-matched children were collected as healthy volunteers during physical examination in our hospitals. For sample preparation, plasma specimens were frozen at −80 °C until use after centrifugation at 400 × *g* for 20 min and 4 °C. Peripheral blood mononuclear cells (PBMCs) were isolated by gradient centrifugation with Ficoll-Hypaque (Cedarlane Laboratories, Hornby, Ontario) according to the manufacturer’s protocols.

### Separation of naive CD4^+^ T cells and platelets

The PBMCs obtained by gradient centrifugation were washed twice with phosphate-buffered (PBS) 500 × *g* for 10 min. According to the instructions of the naive CD4^+^ T cells Isolation Kit II (Miltenyi Biotec, Bergisch Gladbach, Germany), the corresponding Bio-antibody Cocktail II was incubated at 4 °C for 5 min, and MicroBead Cocktail II was incubated at 4 °C for 10 min. It was placed on the MS column of the Midi MACS separator, and then the column was washed twice with 1 mL buffer. The effluent obtained was a naive CD4^+^ T cells suspension. Platelets were isolated from 70 mL of children’s peripheral blood. After centrifugation (1000 r.p.m./min, 10 min, 22 °C), the upper 2/3 of platelet-rich plasma was collected and further centrifuged (500 × *g*, 15 min, 22 °C) in the presence of PBS saline. The platelets were finally resuspended in Dulbecco’s modified Eagle’s medium (Gibco, Grand Island, NY, USA).

### Coculture of naive CD4^+^ T cells with platelets

Separated T cell activation was induced by coated anti-CD3 monoclonal antibody (MAb) (1 μg/mL, 500 μL/well; 3 h, 37 °C) and soluble 1 μg/mL anti-CD28 MAb (all from Invitrogen, Carlsbad, CA, USA). Platelets and CD4^+^ T cells were cultured alone or in combination in RPMI-1640 medium (Biological Industries, Kibbutz Beit Haemek, Israel) containing 10% fetal bovine serum (Gibco, Grand Island, NY, USA), 100 U/mL penicillin, and 100 μg/mL streptomycin (all from Sangon Biotech, Shanghai, China) at naive CD4^+^ T cell–platelet ratios of 1:0, 1:20, 1:40, 1:80, and 1:200. Platelets come from healthy children, mild children, and severe children. Cells were cultured at 37 °C with 5% CO_2_ for up to 3 days.

### Flow cytometry analysis

For cytokine production, the PBMCs were stimulated for 6 h using a Leukocyte Activation Cocktail (1:500) (BD Biosciences, San Jose, CA). The stimulated PBMCs were stained with a BB515-conjugated anti-CD4 antibody at 4 °C for 30 min, and these cells were then fixed and permeabilized using a fixation/permeabilization buffer (BD Biosciences, San Jose, CA). Intracellular staining was performed using allophycocyanin-conjugated anti-IL-4, BB700-conjugated anti-IFN-γ, and phycoerythrin-conjugated anti-IL-17A (both from BD Bioscience) antibodies.

All samples were acquired on a BD Accuri C6 flow cytometer (BD Biosciences), and the data were analyzed using FlowJo software (TreeStar, Ashland, OR, USA).

### RNA extraction and quantitative real-time PCR

To determine the messenger RNA (mRNA) expression levels of the transcription factor T-bet, total RNA was extracted from PBMCs with an RNAprep Pure Cell/Bacteria Kit (TIANGEN, Beijing, China). Then, complementary DNA was synthesized with FastQuant RT Super Mix (TIANGEN, Beijing, China), according to the manufacturer’s protocol. Quantitative polymerase chain reaction (qPCR) was performed in technical triplicate using ChamQ Universal SYBR qPCR Master Mix (Vazyme, Nanjing, China) with a LightCycler® 96 Analysis System (Roche, Basel, Switzerland)). The thermocycler program was as follows: 30 s at 95 °C for denaturation and then 40 cycles (10 s at 95 °C and 30 s at 60 °C) for PCR amplification, followed by amplicon melting analysis to evaluate the specificity of the reaction and identify the presence of primer dimers. All primers were purchased from GenScript Biotech Corp. The primers were as follows: T-bet: sense, 5′-TGT GGG ACA TGG GAG CAG GA-3′, antisense, 5′-CCA GGC CAG CTG TCC AAA GT-3′; GATA-3: sense, 5′-GCC CCT CAT TAA GCC CAA G-3′, antisense, 5′-TTG TGG TGG TCT GAC AGT TCG-3′; ROR-γt: sense, 5′-TGA GAA GGA CAG GGA GCC AA-3′, antisense, 5′-CCA CAG ATT TTG CAA GGG ATC A-3′; CD40: sense, 5′-ACA TAC AAC CAA ACT TCT CCC CG-3′, antisense, 5′-GCA AAA AGT GCT GAC CCA ATC A-3′; Mac-1: sense, 5′-GCC TTG ACC TTA TGT CAT GGG-3′, antisense, 5′-CCT GTG CTG TAG TCG CAC T-3′; PSGL-1 (P-selector glycoprotein ligand-1): sense, 5′-TGT TGC TGA TCC TAC TGG GC-3′, antisense, 5′-CAC AGT GGT AGA CTC AGG GGT-3′, and β-actin: sense, 5′-GTC TCC TCT GAC TTC AAC AGC G-3′; antisense, 5′-ACC ACC CTG TTG CTG TAG CCA A-3′. The RNA expression level of each gene of interest was normalized to that of β-actin, and the results were expressed using the 2–ΔΔCt method.

### Analysis of plasma cytokines

According to the manufacturer’s instructions, the levels of IFN-γ, IL-4, and IL-17A were determined by using a specific ELISA Kit (MultiSciences, Hangzhou, China). All cytokines were quantified using the specific standard curve of recombinant cytokines provided by the corresponding enzyme-linked immunosorbent assay kit.

### Statistical analysis

Statistical comparisons between groups were evaluated by Student’s unpaired (two-tailed) *t* tests. The Spearman’s test was used to analyze the correlation between two continuous variables. All statistical analyses were performed using GraphPad Prism software version 7.0 (GraphPad Software, Inc., La Jolla, CA, USA). A *P* value < 0.05 (two-tailed) was considered statistically significant and labeled with an asterisk. *P* values < 0.01 are labeled with double asterisks, and *P* values < 0.001 are labeled with three asterisks.

## Results

### Clinical characteristics of EV71-infected HFMD patients

Most HFMD is typically characterized by a self-limiting course and good prognosis, but EV71 infection is often predominantly attributed to serious central nervous system complications. This study included 53 EV71-infected HFMD children confirmed by clinical symptoms and results of laboratory tests. Both mild and severe patients had fever and vesicles in the mouth, hands, and feet. There were no significant differences in sex, age, peak fever, or fever duration between the mild and severe groups; however, the duration of vesicles before sample collection was significantly higher in the severe group (3.11 ± 0.29 days) than in the mild group (1.61 ± 0.25 days), suggesting that their clinical staging was nervous system involvement and eruption, respectively. The absolute numbers of lymphocytes were notably increased in children with HFMD compared to healthy controls, but it was conserved in mild patients compared to severe patients. No significant differences in the levels of white blood cell (WBC), neuron-specific enolase (NSE), plateletcrit (PCT), creatine kinase-myocardial band, aspartate aminotransferase, lactate dehydrogenase, albumin, or prognostic nutritional index were identified between the mild and severe groups. The absolute numbers of B cells and the levels of immunoglobulin M (IgM) and its specific antibody EV71-IgM were significantly elevated in severe children with VEM, supporting a significant role for B cells in the clearance of EV71. In addition, the absolute numbers of CD8^+^ T cells increased dramatically in HFMD children compared with healthy children, and the frequency of CD8^+^ T cells indicated a significant increase in the severe group compared with the mild group, suggesting that CD8^+^ T cells play a vital role in anti-EV71 immunity.

### Increased platelet counts in children with EV71 infection

**Fig. 1:**
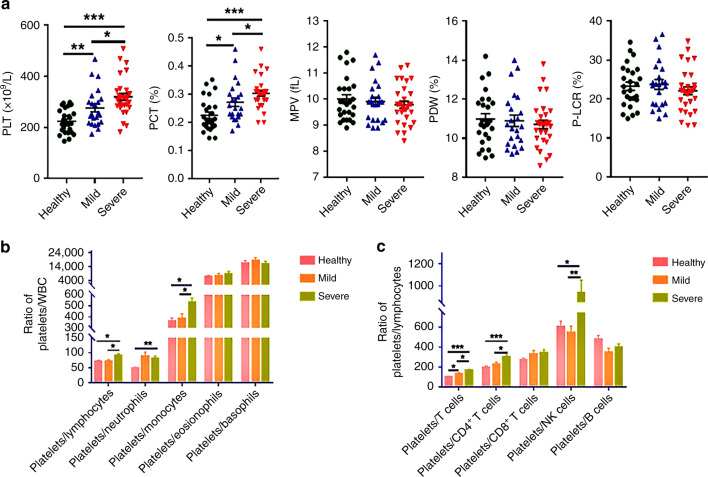
The levels of platelet parameters and platelet-associated inflammatory index parameters in mild HFMD (*n* = 23), severe HFMD (*n* = 30), and healthy controls (*n* = 27). **a** The levels of platelet parameters PLT, PCT, MPV, PDW, and P-LCR were analyzed in mild HFMD, severe HFMD, and healthy controls. **b** The levels of platelets/lymphocytes, platelets/neutrophils, platelets/monocytes, platelets/eosinophils, and platelets/basophils were analyzed in healthy controls, mild HFMD, and severe HFMD. **c** The levels of platelets/T cells, platelets/CD4^+^ T cells, platelets/CD8^+^ T cells, platelets/NK cells, and platelets/B cells were detected in the CFS of mild HFMD, and severe HFMD. Statistical significance was analyzed by Student’s *t* test and one-way ANOVA. *p < 0.05, **p < 0.01, and ***p < 0.001; error bars show the standard deviation. PLT: platelet count; MPV: mean platelet volume; PCT: plateletocrit; PDW: platelet distribution width; P-LCR: platelet large cell ratio (P-LCR).

Generally, platelet function and bioactivity can be assessed by platelet parameters, including PLT, mean platelet volume (MPV), PCT, platelet distribution width (PDW), and platelet large cell ratio (P-LCR), which are easily determined by hematology analysis. PLT and PCT were remarkably higher in both the severe and mild groups than in healthy controls, and severe patients showed increased levels of PLT compared to those in the mild group, suggesting that platelets were involved in the progression of severe HFMD accompanied by VEM (Fig. 1a). No significant alterations were found in MPV, PDW, or P-LCR among all experimental groups (Fig. 1a). The platelet/lymphocyte ratio (PLR) and platelet/monocyte ratio (PLM) increased notably in the severe group compared with the healthy controls and mild group, and the ratio was conserved in the healthy controls and mild group. In addition, the platelet/neutrophil ratio was also higher in severe HFMD children with VEM (Fig. 1b). This result suggested that abnormal inflammatory responses were related to severe neurogenic complications during EV71 infection. Furthermore, as shown in Fig. 1c, children with severe HFMD accompanied by VEM showed a significant increase in the platelet/T cell ratio and platelet/CD4^+^ T cell ratio; however, there were no significant alternations in the platelet/CD8^+^ T cell ratio among all experimental groups, suggesting that platelets were associated with the regulation of CD4^+^ T cell-mediated adaptive immunity.

### Abnormally activated Th1 cells were associated with the onset and progression of EV71 infection

**Fig. 2:**
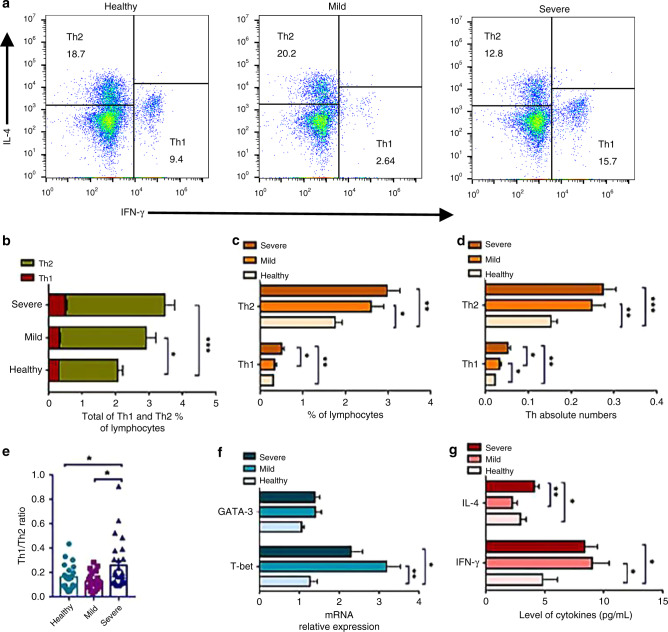
The levels of Th1 and Th2 as well as their associated key transcription factors and effector cytokines in mild HFMD (*n* = 23), severe HFMD (*n* = 30), and healthy controls (*n* = 27). **a** The Th1 and Th2 subpopulations were detected by flow cytometry as indicated with representative examples from mild HFMD, severe HFMD, and healthy controls using the protocol described in the “Methods” section. **b** The total frequencies of circulating Th1 and Th2 were analyzed in healthy controls, mild HFMD, and severe HFMD. **c**–**e** The frequencies and absolute numbers of Th1, Th2, and Th1/Th2 were detected in healthy controls, mild HFMD, and severe HFMD. **f** The levels of T-bet and GATA-3 in healthy controls, mild HFMD, and severe HFMD. **g** The levels of IFN-γ and IL-4 in healthy controls, mild HFMD, and severe HFMD. Statistical significance was analyzed by Student’s *t* test and one-way ANOVA. *p < 0.05, **p < 0.01, and ***p < 0.001; error bars show the standard deviation.

Given the emerging role of platelets in the regulation of CD4^+^ T cell subsets, we sought to analyze Th1 and Th2 cells in the peripheral blood of children with HFMD caused by EV71 (Table 1). The gating strategies and representative plots for each cell subset are reported in Fig. 2a. The total frequency of Th1 and Th2 cells increased significantly in severe patients compared with healthy controls (Fig. 2b). A marked increase in the frequency of Th1 cells was indicated in the severe group compared with that in the mild group and healthy controls (Fig. 2c). To adjust for alterations in lymphocyte counts of each group, the absolute numbers for Th1 and Th2 cells were calculated. In line with a significant increase in the frequency of Th1 cells, the Th1 absolute numbers were substantially elevated in the severe group, and it had a positive correlation with the severity of HFMD (Fig. 2d). In addition, children with HFMD displayed remarkably more absolute numbers for Th2 cells (Fig. 2d) and a higher frequency of the Th cell subset (Fig. 2c). Moreover, compared to healthy controls and mild patients, the Th1/Th2 ratio increased significantly in severe patients, suggesting that severe HFMD children with VEM were skewed toward Th1-medicated anti-EV71 immunity in the periphery (Fig. 2e). To further dissect the activity of Th1 and Th2 cells in children with severe HFMD, the mRNA levels of the transcription factors T-bet and GATA-3 in PBMCs were determined. As shown in Fig. 2f, the level of T-bet was dramatically elevated in both mild and severe patients compared with that in healthy controls. However, no significant changes were identified in GATA-3 mRNA expression among those groups (Fig. 2f). Furthermore, children with HFMD showed an increased level of IFN-γ, but it was comparable in mild patients and severe patients, suggesting that overactivated Th1 cells were related to the onset and progression of EV71 infection (Fig. 2g). The level of IL-4 was significantly increased in severe patients compared with that in mild patients and healthy controls.

**Table 1:** Clinical characteristics of 26 healthy controls and 53 recruited cases with mild and severe HFMD caused by EV71.

Group	Healthy	Mild	Severe
Age (months)	29.96 ± 1.97	27.65 ± 3.33	36.79 ± 2.92
Gender (M/F)	26 (18/8)	23(18/5)	30 (22/8)
Fever >37.5 °C (days)	NA	2.09 ± 0.30	2.70 ± 0.25
Rash (days)	NA	1.61 ± 0.30	3.11 ± 0.29^a^
WBC in PB (×10^9^/L)	8.68 ± 0.18	10.01 ± 0.63^a^	8.89 ± 0.36^b^
Lymphocyte (×10^9^/L)	3.30 ± 0.09	4.21 ± 0.36	3.67 ± 0.14
Monocytes (×10^9^/L)	0.66 ± 0.02	0.81 ± 0.08	0.67 ± 0.04
Neutrophil (×10^9^/L)	4.67 ± 0.12	4.78 ± 0.63	4.45 ± 0.36
Eosinophilia (×10^9^/L)	0.05 ± 0.01	0.11 ± 0.03	0.06 ± 0.01
Basophilic (×10^9^/L)	0.03 ± 0.00	0.02 ± 0.21	0.02 ± 0.01
T cell (×10^9^/L)	2.25 ± 0.08	2.39 ± 0.26	2.23 ± 0.10
CD3^+^CD4^+^ T cell (×10^9^/L)	1.26 ± 0.07	1.36 ± 0.18	1.17 ± 0.07
CD3^+^CD8^+^ T cell (×10^9^/L)	0.87 ± 0.03	0.97 ± 0.10	1.00 ± 0.05
CD4^+^CD8^+^ T cell (×10^9^/L)	0.07 ± 0.01	0.05 ± 0.01	0.05 ± 0.01
NK cell (×10^9^/L)	0.44 ± 0.03	0.62 ± 0.08	0.46 ± 0.04
B cell (×10^9^/L)	0.54 ± 0.03	0.88 ± 0.09	0.86 ± 0.05
CD4^+^/CD8^+^ ratio	1.66 ± 0.14	1.46 ± 0.11	1.18 ± 0.06
Nucleated cell in CSF (×10^9^/L)	NA	3.79 ± 1.23	77.67 ± 9.25^a^
CRP (<8 mg/L)	98%	94%	94%
CK-MB (U/L)	27.23 ± 1.14	26.38 ± 1.96	30.68 ± 3.76
AST (mmol/L)	29.27 ± 1.09	29.18 ± 1.67	30.02 ± 1.61
LDH (U/L)	270.80 ± 5.74	273.4 ± 11.9	281.3 ± 16.4
ALB (g/L)	NA	40.94 ± 0.67	42.34 ± 0.62
NSE (ng/mL)	20.70 ± 0.80	21.46 ± 2.06	23.80 ± 4.00
PCT (ng/mL)	0.06 ± 0.01	0.54 ± 0.10	0.52 ± 0.39
IgG	8.79 ± 0.30	7.59 ± 0.59	9.14 ± 0.61
IgM	1.19 ± 0.04	1.10 ± 0.08	1.37 ± 0.09^a^
IgA	0.93 ± 0.04	0.78 ± 0.16	0.94 ± 0.11
C3	1.14 ± 0.07	1.13 ± 0.07	1.20 ± 0.03
C4	0.31 ± 0.02	0.31 ± 0.05	0.29 ± 0.02
EV71-IgM	NA	0.19 ± 0.14	1.18 ± 0.14^a^

Note: The data correspond to the arithmetic mean ± SEM.

*PB* peripheral blood, *M/F* male/female, *WBC* white blood cell, *CRP* C-reactive protein, *CK-MB* creatine kinase-myocardial band, *AST* aspartate aminotransferase, *LDH* lactate dehydrogenase, *NSE* neuron-specific enolase, *PCT* procalcitonin, *NA* not applicable.

^a^*P < 0.05, severe compared with mild group.

^b^*P  <  0.05, severe compared with healthy group.

### Platelets are involved in the development of VEM by antagonizing the Th1 cell function

**Fig. 3:**
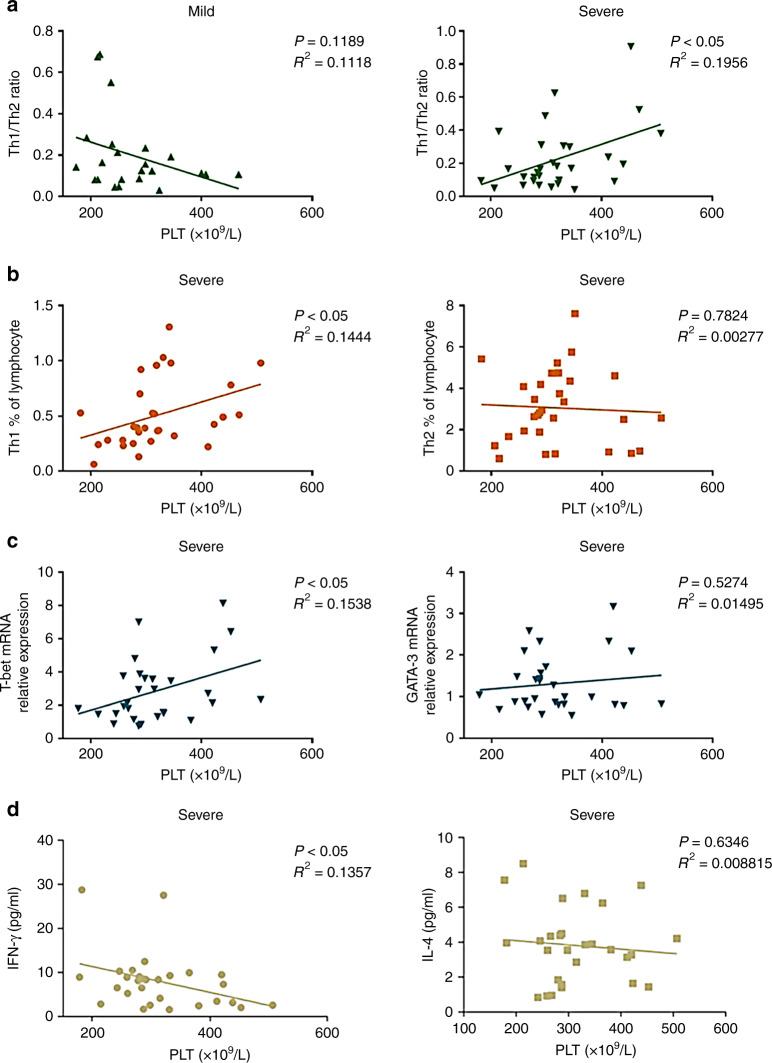
Relationship between PLT and CD4^+^ T cell subset or its associated key transcription factors. **a** The correlations between the levels of PLT and Th1/Th2 ratio are shown in mild and severe patients. **b** The correlation between the levels of PLT and the frequency of Th1 or Th2 is shown in severe patients. **c** The correlation between the levels of PLT and T-bet or GATA-3 is shown in severe patients. **d** The correlation between the level of PLT and IFN-γ or IL-4 is shown in severe patients. Statistical significance was analyzed by Student’s *t* test and one-way ANOVA. *p < 0.05, **p < 0.01, and ***p < 0.001; error bars show the standard deviation. Lines indicate linear regression. Spearman’s rank correlation coefficients (*R*^2^) and the corresponding *p* values are indicated. PLT: platelet count.

Numerous recent studies have pointed out that platelets play a major role in CD4^+^ T cell subset differentiation and activation; however, the effect of platelets on the function of Th1 and Th2 cell subsets in the onset and progression of EV71 infection remains unclear. We first discovered that the level of PLT was positively correlated with the Th1/Th2 ratio in children with severe HFMD, and there was no significant correlation between them in children with mild HFMD (Fig. 3a), suggesting that platelets have a crucial function in the Th1/Th2 balance in HFMD children with VEM. In addition, to further dissect the association between PLT and the Th1/Th2 ratio in severe patients, we analyzed the relationship between the frequencies of Th1 or Th2 cells and the level of PLT in severe patients. Correlation analysis demonstrated that there was a clear positive correlation between the level of Th1 cells and PLT, and there was no clear relation between Th2 cells and PLT (Fig. 3b), supporting important roles for platelets in promoting Th1 cell differentiation. Furthermore, as shown in Fig. 3c, the level of T-bet mRNA expression was positively affected by platelets. Interestingly, we found a significant negative correlation between the inflammatory factor IFN-γ and platelets in severe HFMD patients; however, the expression of IL-4 had a downward trend, but it was not obvious (Fig. 3d), suggesting that platelets could block IFN-γ secretion while promoting Th1 differentiation.

### Differential roles of platelets in the regulation of Th1 cell differentiation and function in vitro

**Fig. 4:**
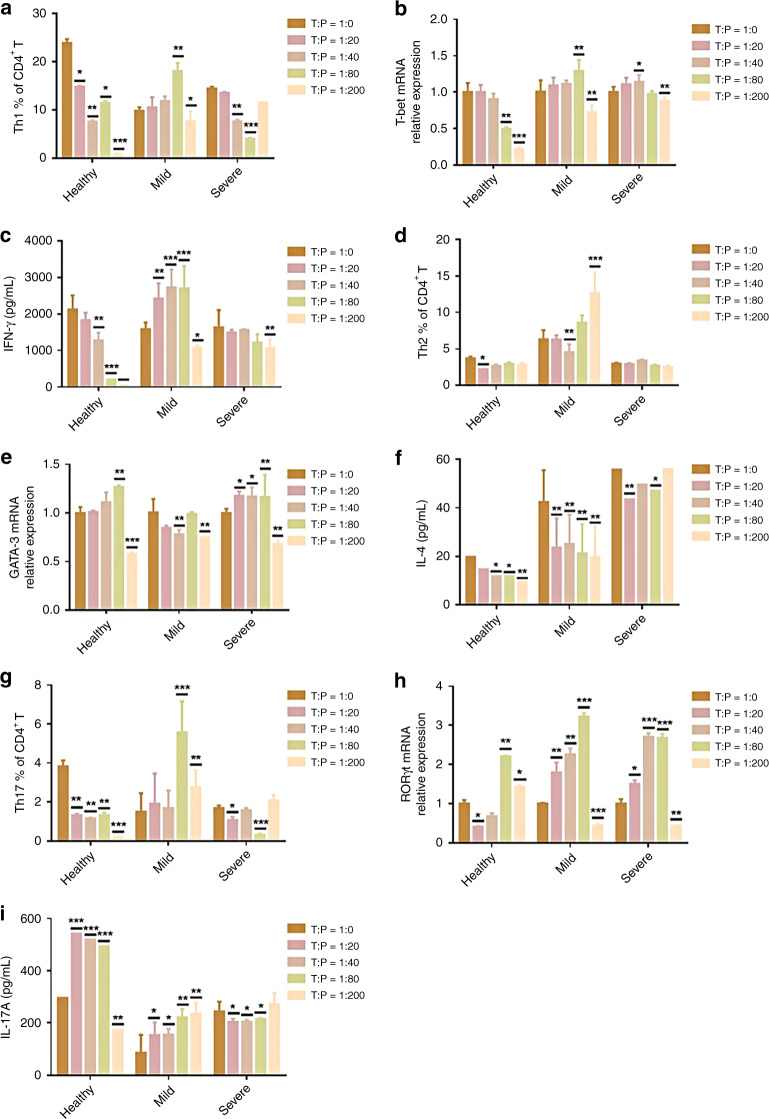
In the case of different naive CD4^+^ T:platelets (T/P), the platelets of mild, healthy, and severe children with naive CD4^+^ T cells of healthy children were incubated in vitro for 3 days. **a**–**c** The level of Th1 was detected by FACS, the levels of IFN-γ were detected by ELISA, and the level of T-bet mRNA relative expression was detected by qPCR. **d**–**f** The level of Th2 was detected by FACS, the levels of IL-4 were detected by ELISA, and the level of GATA-3 mRNA relative expression was detected by qPCR. **g**–**i** The level of Th17 was detected by FACS, the levels of IL-17A were detected by ELISA, and the level of RORγt mRNA relative expression was detected by qPCR. Statistical significance was analyzed by Student’s *t* test and one-way ANOVA. *p < 0.05, **p < 0.01, and ***p < 0.001; error bars show the standard deviation.

To further verify that platelets from severe patients inhibit Th1 cell function, we carried out a platelet-naive CD4^+^ T cell coculture experiment in vitro. With the increased ratio of platelet-to-naive CD4^+^ T cells (P:T), the frequency of Th1 cells in the severe group decreased significantly, as did the levels of T-bet mRNA expression and IFN-γ, while in the mild group, there was a significant increase (Fig. 4a–c). However, platelets had little effect on the differentiation of Th2 cells, the mRNA expression of GATA-3, and the production of cytokine IL-4 (Fig. 4d–f). On the other hand, Th17 and RORγt mRNA expression as well as their cytokine IL-17A decreased in the severe group and increased in the mild group (Fig. 4g–i), suggesting that platelets could block the differentiation and function of Th1 and Th17 cells in the later stage of inflammation and play a proinflammatory role. Interestingly, when the concentration ratio reached 1:200, the relevant indicators decreased, and we suspect that when the platelet count increases to a certain extent; it may begin to play an anti-inflammatory role, which needs to be further verified. The trend in the healthy group was opposite to that in the mild group (Fig. 4a–c, g–i). With an increase in the platelet ratio, the number of Th1 and Th17 cells decreased, the level of IFN-γ decreased, and the level of Th2 cells and its cytokine IL-4 decreased slightly; however, the change was not obvious (Fig. 4d–f), suggesting that platelets may be the protective mechanism of the body in the normal state.

### Platelets blocked the function of Th1 cells by directly contacting naive CD4^+^ T cells

**Fig. 5:**
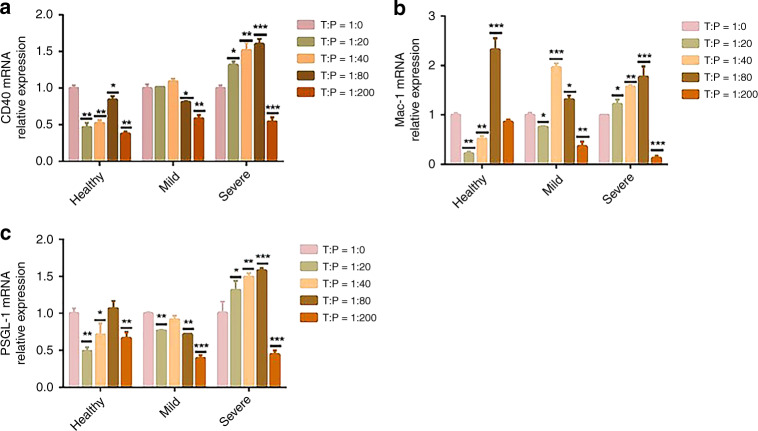
Under the condition of different naive CD4^+^ T:platelets (T/P), platelets and naive CD4^+^ T cells were incubated in vitro for 3 days. **a** The level of CD40 mRNA relative expression is shown in healthy, mild, and severe patients. **b** The level of Mac-1 mRNA relative expression is shown in healthy, mild, and severe patients. **c** The level of PSGL-1 mRNA relative expression is shown in healthy, mild, and severe patients. Statistical significance was analyzed by Student’s *t* test and one-way ANOVA. *p < 0.05, **p < 0.01, and ***p < 0.001; error bars show the standard deviation.

To explore the mechanism of the differential roles of platelets in the regulation of pathogenic CD4^+^ T cell differentiation and function, we detected the mRNA levels of the T cell system. As shown in Fig. 5a, compared with the T cells cultured alone, with the increase in the P:T, the gene expression of the T cell surface molecule CD40 decreased at first and then increased in the healthy group. In the mild group, gene expression decreased with the increase in P:T, while in the severe group, gene expression increased gradually within a certain range of platelet concentrations (Fig. 5a). Compared with the cultured T cells alone, leukocyte integrin Mac-1, which interacted with platelet surface GPIbα, had a significant upward trend in the severe group, but only increased when the ratio was 1:80 in the healthy group. In the mild group, there was a significant increase at 1:40 and 1:80 (Fig. 5b). The gene expression of PSGL-1, which binds to P-selectin on platelets, decreased gradually in mild patients and increased significantly in severe patients (Fig. 5c). We preliminarily concluded that platelets in severe patients mainly regulate T cells through CD40L, GPIbα, and CD62P contacting T cells. However, the mode of action of platelets in patients with mild HFMD needs to be further verified.

## Discussion

Emerging studies have demonstrated that more than a key mediator of hemostasis, platelets are also increasingly recognized as crucial immune modulatory cells by recognizing RAMP (resolution-associated molecular pattern) or DAMP (damage-associated molecular pattern) and communicating with T cells.^26^ Moreover, it has been evident from recent studies that platelets are involved in the regulation of the inflammatory response caused by viral infection.^27^ However, the potential impact of platelets on EV71 infection is not yet known. In the present study, we found that platelet counts were positively associated with the severity of HFMD infection caused by EV71 (Fig. 1a), thus revealing that platelets play vital roles in the onset and progression of EV71 infection. In addition, the PLR and PLM displayed a significant increase in the children with severe HFMD compared to mild patients and healthy volunteers (Fig. 1b), suggesting that severe patients with VEM complications exhibit excessively activated inflammatory responses. Given that platelets may serve as immune regulatory cells that affect all stages of antiviral immunity, we speculated that platelets may function as major inflammatory cells involved in the regulation of CD4^+^ T cell subsets from anti-EV71 immunity. As previous studies indicated, EV71 infection contributed to an imbalance between Th1 and Th2 cells as well as the associated cytokines IFN-γ and IL-4. In addition, the Th17/Treg ratio level was evaluated significantly in severe patients with VEM complications. In our study, in line with previous studies, we observed that severe patients display higher levels of Th1 cells than those in mild and healthy volunteers (Fig. 2c), and the absolute number of Th1 cells was positively correlated with the severity of EV71 infection (Fig. 2d). In addition, there was a significant increase in Th2 cells in children with HFMD compared to healthy volunteers (Fig. 2c, d). Moreover, the Th1/Th2 ratio was increased significantly in severe patients compared to healthy volunteers and mild patients, suggesting that abnormally activated Th1 cells are implicated in the pathogenesis of EV71 infection (Fig. 2e). Recently, platelets were discovered to play key roles in the regulation of CD4^+^ T cell subset differentiation and activation. For example, platelets promoted the differentiation of Th1 and Th17 cells in vivo. To study whether platelets contribute to Th1 cell polarization, we observed that patients with higher platelet levels showed a larger number of Th1 cells (Fig. 3b), which was also confirmed by in vitro coculture experiments (Fig. 4a). In addition, there was a positive correlation between the platelet level and Th1/Th2 ratio in severe patients (Fig. 3a), suggesting that platelets can promote Th1 cell differentiation in patients with severe VEM complications, but the level of IFN-γ in severe patients was lower than that in mild patients and was negatively correlated with platelets (Figs. 2g, 3d). In vitro, we found that platelets from severe patients inhibited Th1 cell differentiation with increasing platelet concentration (Fig. 4a). Therefore, we hypothesized that platelets in severe patients could block the function of Th1 cells. The results of in vitro experiments supported this hypothesis. Together, combined with literature reports^15^ and coculture experiments of platelet-naive CD4^+^ T cells in vitro, we found that platelets could inhibit the differentiation of Th1 and Th17 cells and related functions in severe patients, but in the mild group, platelets could promote the differentiation of Th1 cells and their expression of IFN-γ (Fig. 4a, c, g, i). There are two main ways that platelets and T cells interact.^15^ Platelet α-particles and dense particles store various proinflammatory and regulatory mediators, which are rapidly released at the site of inflammation or tissue injury. In addition to secreting soluble factors, activated platelets also upregulate different integrins, adhesion molecules and lectins to form platelet–T cell aggregates and then inhibit the proliferation of T cells. In our study, we found that several typical molecules expressed on T cells that bind to platelets, including leukocyte integrin Mac-1, interact with GPIbα on the surface of platelets.^28^ Our results showed that compared to healthy volunteers and mild patients, the expression of mRNA of related molecules increased significantly with an increase in the platelet number in severe patients (Fig. 5a–c), indicating that platelets combined with T cells to inhibit the functions of T cells. Based on the present results, it is reasonable to speculate that platelets initially promote the differentiation of Th1 cells in the early stage of EV71 infection. When the number of platelets and T cells increased in the late stage of HFMD, platelets directly contacted T cells to inhibit the proliferation of T cells. However, whether platelets can promote the differentiation of Th1 cells through the secretion of cytokines in mild patients remains to be further investigated.

## Conclusion

This study showed that EV71 infection leads to an elevation in platelets, resulting in Th1 cell polarization in mild patients, whereas platelets block Th1 cell differentiation and function in severe patients, suggesting that EV71-infected platelets play distinct roles in the regulation of pathogenic CD4^+^ T cells in the onset and development of HFMD. In addition, platelets could directly contact T cells to form aggregates to inhibit the differentiation and functional expression of Th1 cells in severe infection. The present study provides new insights into the pathogenesis of HFMD infected by EV71 and offers a potentially new strategy for the intervention of this disease at different stages.
